# *Ornithodoros* (*Pavlovskyella*) ticks associated with a *Rickettsia* sp. in Pakistan

**DOI:** 10.1186/s13071-022-05248-0

**Published:** 2022-04-21

**Authors:** Abid Ali, Muhammad Numan, Mehran Khan, Ome Aiman, Sebastián Muñoz-Leal, Lidia Chitimia-Dobler, Marcelo B. Labruna, Ard M. Nijhof

**Affiliations:** 1 Department of Zoology, Abdul Wali Khan University Mardan, Mardan, Khyber Pakhtunkhwa Pakistan; 2 Departamento de Ciencia Animal, Facultad de Ciencias Veterinarias, Universidad de Concepción, Chillán, Ñuble Chile; 3 Bundeswehr Institute of Microbiology, Munich, Germany; 4 Department of Preventive Veterinary Medicine and Animal Health, Faculty of Veterinary Medicine, University of São Paulo, São Paulo, Brazil; 5 Institute for Parasitology and Tropical Veterinary Medicine, Freie Universität Berlin, Berlin, Germany

**Keywords:** Ticks, Argasidae, *Ornithodoros*, *Rickettsia*, Pakistan

## Abstract

**Background:**

Soft ticks (Ixodida: Argasidae) are medically important ectoparasites that mainly feed on birds and mammals, which play a key role in their geographic distribution and dispersion. Despite their importance, studies on soft ticks are scarce for many regions and countries of the world, including Pakistan.

**Methods:**

In this study, 2330 soft ticks—179 larvae (7.7%), 850 nymphs (36.4%), 711 males (30.5%) and 590 females (25.3%)—were collected from animal shelters in 18 locations within five districts of Khyber Pakhtunkhwa, Pakistan. A subset of the collected ticks was processed for DNA extraction and polymerase chain reaction (PCR) for the amplification of tick 12S ribosomal DNA (rDNA), 16S rDNA and cytochrome c oxidase subunit I (*cox1*), and rickettsial 16S rDNA gene fragments. The obtained sequences were used for the construction of a phylogenetic tree.

**Results:**

All the specimens were morphologically identified as *Ornithodoros*, and were morphologically similar to *Ornithodoros tholozani*. The genus was confirmed by sequencing partial 12S rDNA, 16S rDNA and *cox1* gene fragments. Additionally, a *Rickettsia* sp. was detected in some of the collected ticks by PCR targeting 16S rDNA. The morphological relatedness of the tick specimens with *O. tholozani* was confirmed by phylogenetic analysis, in which the *Ornithodoros* sp. clustered with *Ornithodoros tholozani* and *Ornithodoros verrucosus*, both of which belong to the subgenus *Pavlovskyella* and have been previously reported from Israel, Ukraine and Iran. The phylogenetic tree also indicated that the *Ornithodoros* sp. from Pakistan corresponds to an undetermined species. Furthermore, the associated *Rickettsia* sp. grouped with the limoniae group of *Rickettsia* species previously reported from *Argas japonicus* ticks from China.

**Conclusions:**

This is the first molecular study of an *Ornithodoros* species from Pakistan. Further studies are essential to confirm its identity and possible pathogenicity with regard to its associated microorganisms in the studied region.

**Graphical abstract:**

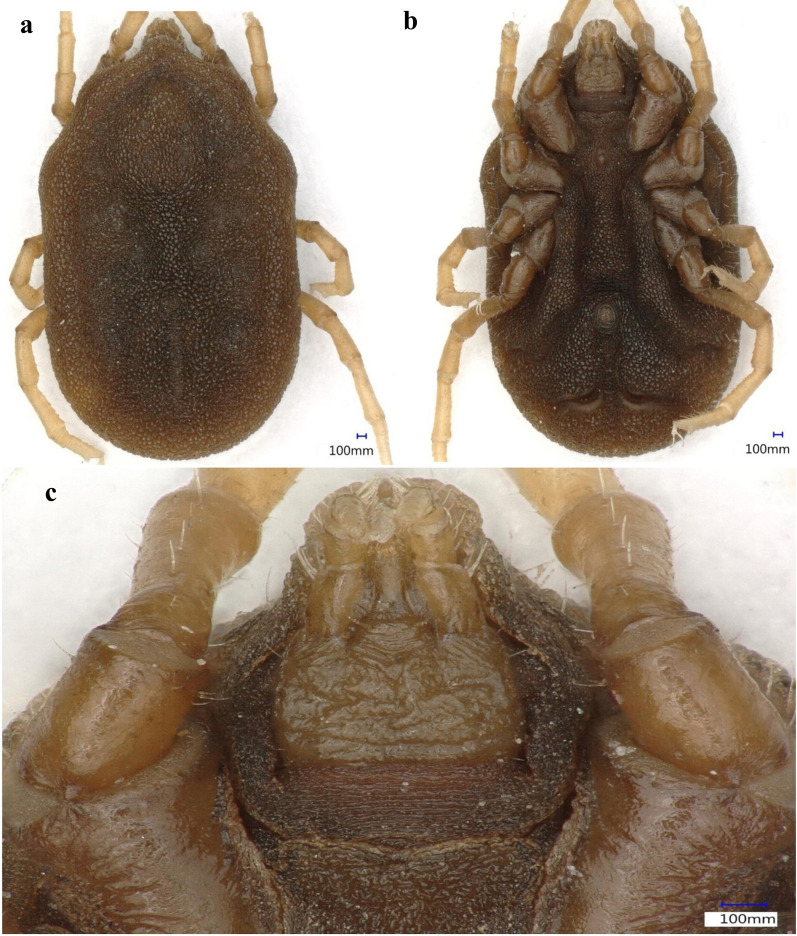

## Background

Soft ticks (Ixodida: Argasidae) are medically important hematophagous ectoparasites that feed on terrestrial vertebrates, mostly birds and mammals [1, 2]. Among the Argasidae, some species are host specific, while others are generalists and feed on a variety of hosts when encountered in their microhabitats [3]. They are distributed throughout the world and spread to new geographic regions through the movement of their hosts, such as bats and migratory birds [4, 5]. Soft ticks have an endophilic or nidicolous lifestyle and are found in sheltered habitats such as burrows, cracks, crevices, nests and loose soil [3, 6, 7]. Fewer studies have been conducted on soft ticks than on hard ticks (Ixodidae), likely because of the former’s nidicolous lifestyle and short feeding duration, which make these parasites difficult to observe in the field [6].

The family Argasidae comprises 218 species, of which approximately 133 have not been assigned accurately to genera [8, 9]. *Ornithodoros* is the most diverse genus within the Argasidae, with approximately 130 known species [2, 10–13]. The phylogeny and taxonomy of argasid ticks are still controversial, and many species have been assigned to more than one genus [14]. In this study, we follow the nomenclature proposed by Mans et al. [15] who, among others, showed that phylogenetic reconstructions using tick mitogenomes indicate that the genus *Ornithodoros* (*Pavlovskyella*) is paraphyletic [15]. *Ornithodoros tholozani* has been described from specimens collected in Persia [16], a geographical region that does not include Pakistan. Only two *Ornithodoros* species, *Ornithodoros tholozani* [17, 18] and *Ornithodoros papillipes* [19], which were identified morphologically, have been reported in Pakistan thus far. *Ornithodoros tholozani* mainly infests livestock [17], birds and pigs [4]. A similar tick, *Alveonasus lahorensis*, also parasitizes cattle, camels and sheep, although the Asiatic mouflon is its primary host [4]. In Pakistan, *O. tholozani* is widely distributed, whereas the distribution of *A. lahorensis* is limited to the western part of the country [20]. Currently, no genetic data are available for *Ornithodoros* spp. from Pakistan.

Soft ticks are considered to be reservoirs for several arboviruses, including African swine fever virus, as well as relapsing fever, which is caused by species of the genus *Borrelia*, and several *Rickettsia* species [21–23]. Some *Rickettsia* species are endosymbionts, whereas others are pathogens of vertebrate hosts, including humans [24]. Depending on their genotypical and phenotypical characteristics, the rickettsiae have been divided into four major groups, namely the spotted fever, typhus, bellii, and limoniae groups [25]. Further, several *Rickettsia* species have been reported from *Ornithodoros* species, such as “*Candidatus* Rickettsia africaseptentrionalis” and “*Candidatus* Rickettsia mauretanica” [26], “*Candidatus* Rickettsia wissemanii*”* [27], *Rickettsia hoogstraalii* [28], *Rickettsia lusitaniae* [28], “*Candidatus* Rickettsia nicoyana*”* [29], and many others that have yet to be identified to species level [24].

The traditional method of tick identification is based on morphological traits and requires a specialist’s expertise, particularly for soft ticks. The morphological identification of argasid ticks is particularly difficult in the case of closely related species, immature stages, engorged stages and damaged specimens [30]. The identification of argasid ticks can be facilitated by the use of genetic markers, such as 12S ribosomal DNA (rDNA), 16S rDNA and cytochrome c oxidase subunit I (*cox1*) mitochondrial genes [31–33]. Moreover, there is a lack of data on the *Rickettsia* species harbored by soft ticks in Pakistan [17, 31, 34–36]. Therefore, the aims of this study were to genetically identify soft ticks collected in the mountainous region of Khyber Pakhtunkhwa (KP) in Pakistan, and to detect the presence of *Rickettsia* DNA in these ticks.

## Methods

### Study area

**Fig. 1:**
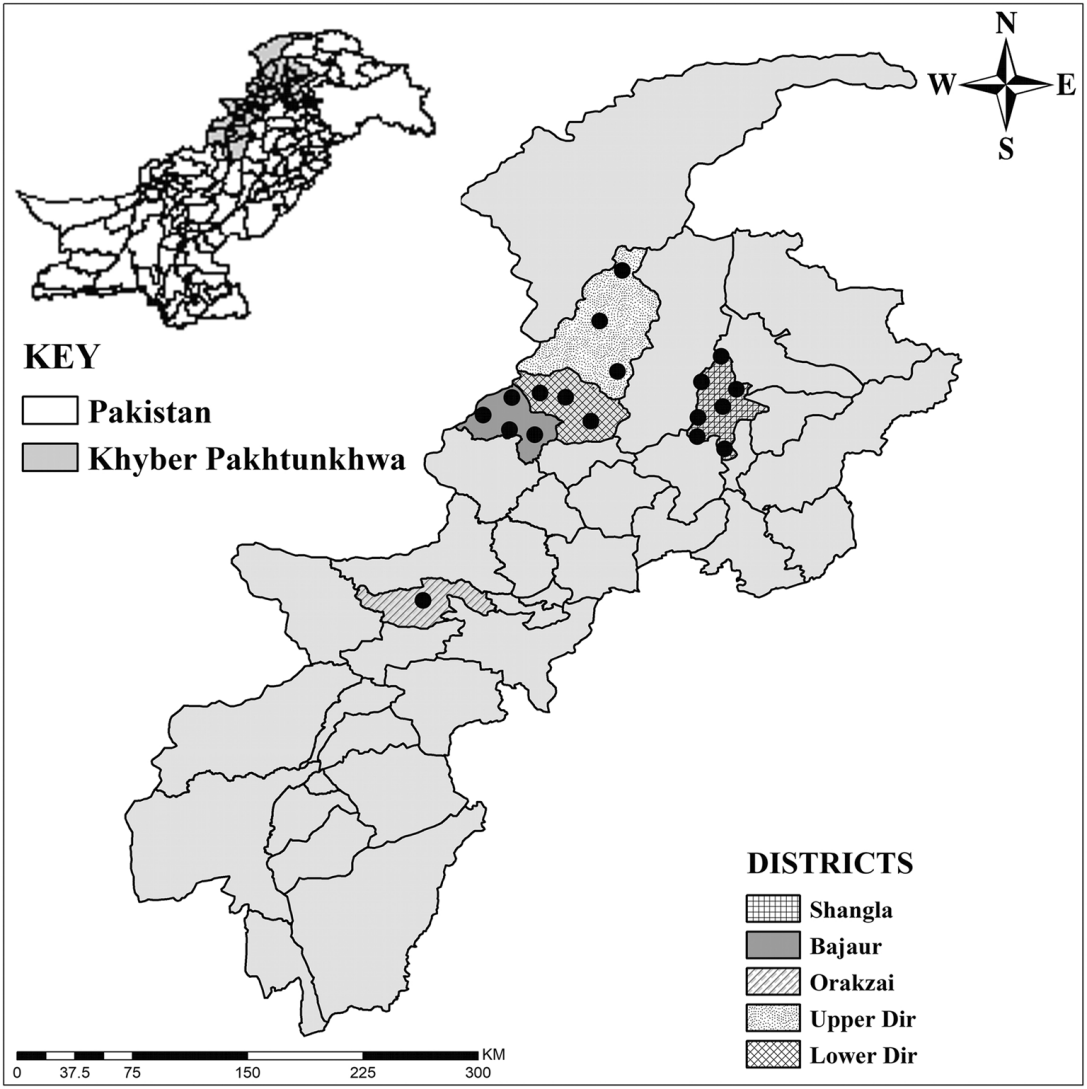
Map showing the 18 locations (black dots) where the soft ticks were collected

Khyber Pakhtunkhwa (KP) province is in northwestern Pakistan, adjacent to Afghanistan, with Baluchistan province to the south, Punjab province to the east and Azad Jammu Kashmir and Gilgit provinces to the northeast. The present study was conducted in the following five districts of KP: Shangla (34°52'50.6"N, 72°35'27.0"E), Bajaur (34°43'56.1"N, 71°30'33.1"E), Lower Dir (34°52'12.1"N, 71°49'00.8"E), Upper Dir (35°20'28.7"N, 72°03'40.0"E), and Orakzai (33°41'21.3"N, 70°57'26.6"E). These districts are located in mountainous territory at high elevation. The highest elevation, annual average temperature, relative humidity and precipitation of these districts are as follows: Shangla—3440 m, 14 °C, 67% and 850 mm; Bajaur—1150 m, 21 °C, 64% and 700 mm; Lower Dir—1340 m, 17 °C, 68% and 730 mm; Upper Dir—1860 m, 15 °C, 65% and 770 mm; and Orakzai—2500 m, 25 °C, 65% and 600 mm. Google Maps was used to find the geographical coordinates of each location, and the study map (Fig. 1) was designed using ArcGIS 10.3.1.

### Tick collection and preservation

Ticks were collected manually from crevices, cracks, burrows and debris in 60 different animal shelters (housing cows, buffaloes, goats, sheep, and equids) in a total of 18 sampling locations in the selected districts (Fig. 1). Tick collection was performed during the day and at night between June and October 2019. The tick samples were stored in Eppendorf tubes and transported to the Department of Zoology, Abdul Wali Khan University, Mardan. Some of the collected ticks were subjected to low intensity ultrasonic vibration for 5 min with a Qsonica sonicator Q700, LLC (Newtown, CT), and washed with distilled water and 70% ethanol to remove contaminants. The specimens were subjected to morphological identification and individually preserved in 100% ethanol in 2-ml Eppendorf tubes for further molecular examination.

### Morphological identification

The collected soft ticks were morphologically identified to genus level using a standard identification key [37]. Representative specimens were photographed at 50-200× magnification using a Keyence VHX 900F microscope (Keyence, Itasca, IL).

### DNA extraction and PCR

A total of 54 specimens, i.e. two nymphs and one female from each of the 18 sampling locations, were subjected to DNA extraction for subsequent genetic identification. Each specimen was cut in half using a sterile blade and the DNA of one half was extracted using the Nucleospin Tissue Kit (Macherey-Nagel, Düren, Germany) according to the manufacturer’s guidelines. Extracted DNA was stored at −20 °C until further analysis.

Extracted DNA samples were examined by PCR amplification of partial fragments of the 12S rDNA, 16S rDNA and *cox1* tick mitochondrial genes. A 25-µl PCR reaction mixture was prepared for 16S rDNA and *cox1* genes; the mixture consisted of 12.75 µl water, 5 µl of 5X Fusion HF Buffer (Mobidiag), 2.5 µl of 2 mM deoxyribose nucleoside triphosphates (dNTPs), 1 µl of 10 µM primers, 0.25 S7 Fusion polymerase and 2.5 µl template DNA. The primers 16S+1 (5’-CCGGTCTGAACTCAGATCAAGT-3’) and 16S-1 (5’-GCTCAATGATTTTTTAAATTGCTGT-3’) were used for the amplification of 16S rDNA fragments [38]. Universal *cox1* gene primers HC02198 (5’-TAAACTTCAGGGTGACCAAAAAATCA-3’) and LCO1490 (5’-GGTCAACAAATCATAAAGATATTGG-3’) were used for the amplification of fragments of the *cox1* gene [39]. Cycling conditions were as follows: 98 °C for 30 s, followed by 40 cycles of 98 °C for 10 s, 55 °C (16S rDNA) or 63 °C (*cox1*) for 20 s, 72 °C for 15 s (16S rDNA) or 25 s (*cox1*), and a final extension step at 72 °C for 5 min. For the amplification of 12S rDNA fragments, PCR was performed in a 25-µl reaction mixture containing 2.5 µl 10X DreamTaq Buffer (Thermo Fisher Scientific), 2.5 µl 2 mM dNTPs, 1 µl of 10 µM primers SR-J-14199 (5’-TACTATGTTACGACTTAT-3’) and SR-N-14594 (5’-AAACTAGGATTAGATACCC-3’) [40], 15.375 µl water, 0.125 (5 U/μl) of DreamTaq polymerase (Thermo Fisher Scientific) and 2.5 µl DNA template. Cycling conditions were as follows: 95 °C for 3 min, followed by 40 cycles of 95 °C for 30 s, 50 °C for 30 s, 72 °C for 25 s, and finally 72 °C for 5 min.

PCR reaction mixtures (25 µl) for the amplification of fragments of the 16S rDNA gene of *Rickettsia* species consisted of 12.75 µl water, 5 µl of 5X Fusion HF buffer (Mobidiag), 2.5 µl of 2 mM dNTPs, 1 µl of 10 µM primers Eh-out1 (5’-TTGAGAGTTTGATCCTGGCTCAGAACG-3’) and Ehr3-17 (5’-TAAGGTGGTAATCCAGC-3’) [41], 2.5 µl DNA template and 0.25 µl S7 Fusion polymerase. Cycling conditions were as follows: 98 °C for 30 s, followed by 35 cycles of 98 °C for 10 s, 55.9 °C for 15 s, 72 °C for 10 s, with a final extension at 72 °C for 10 min. The PCR reactions contained *Argas persicus* DNA as the positive control in the case of tick species, and *Rickettsia massiliae* DNA as the positive control for *Rickettsia* species, while PCR-grade water was used as the negative control. The PCR amplified products were run on a 2% agarose gel and observed using gel documentation (BioDoc-It Imaging Systems). Amplicons were purified using the DNA Clean & Concentrator Kit (Zymo Research, Irvine, CA) and sequenced in both directions by LGC Genomics (Berlin, Germany).

### Sequencing and phylogenetic analysis

The obtained sequences were trimmed using SeqMan version 5.0 (DNASTAR) to remove primers and poor-quality sequence reads, and subjected to a Basic Local Alignment Search Tool [BLASTn; National Center for Biotechnology Information (NCBI)] search. Trimmed sequences were imported together with sequences from related species in BioEdit alignment editor v 7.0.5 [42] and subjected to ClustalW Multiple alignment [43]. The phylogenetic trees for tick mitochondrial 12S rDNA, 16S rDNA, and *cox1* sequences, and for *Rickettsia *16S rDNA sequences, were created separately, in accordance with the neighbor-joining method in Molecular evolutionary genetics analysis (MEGA-X) software [44], using align by MUSCLE [45] with a bootstrapping value of 1000 [44].

## Results

### Identified ticks

**Fig. 2:**
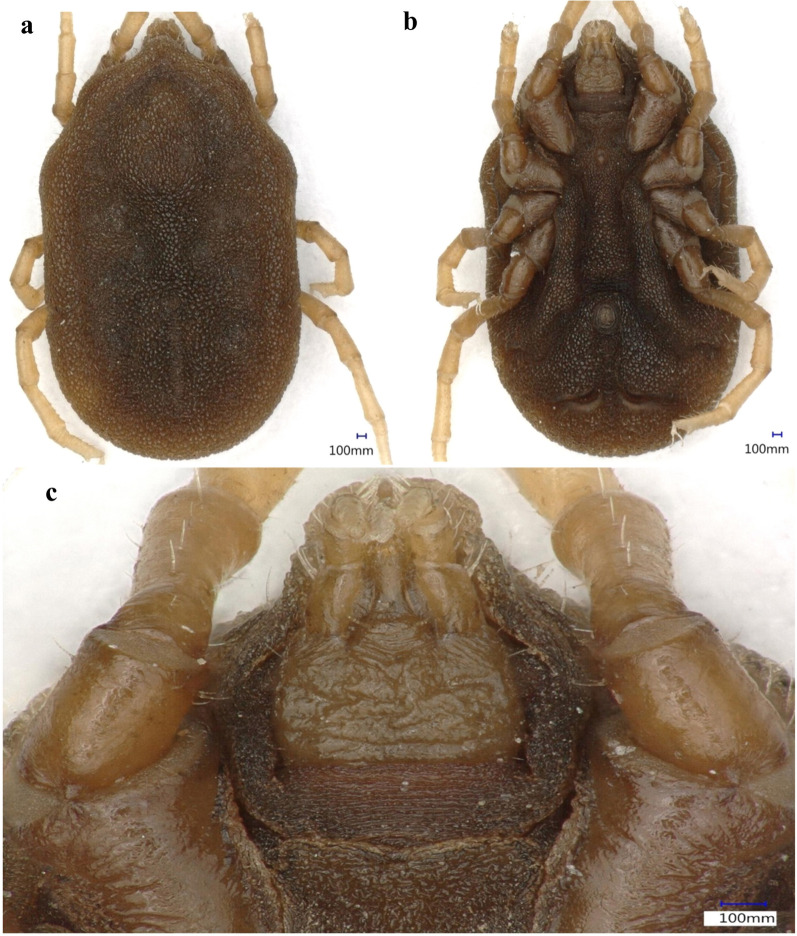
Dorsal (**a**), ventral (**b**) and capitulum (**c**) views of a nymph of the *Ornithodoros* sp. collected in this study

In total, 2330 soft ticks were collected (Table 1) and morphologically identified as *Ornithodoros* (*Pavlovskyella*) sp. from the following traits: nymphs and adults had small cheeks; the dorsal idiosome had faint dorsal disks and small mammillae; a dorsoventral groove was present; a pre-anal groove, which completely intersected the transverse postanal groove, was present (Fig. 2).

**Table 1:** Locations and probable associated hosts of the *Ornithodoros* sp. collected in this study, and the presence of *Rickettsia* DNA detected by polymerase chain reacion

Districts	Localities	Month and year of collection	Collection points (inside animal shelters)	Probable associated hosts	Ticks submitted for molecular analysis	*Rickettsia* sp. detected
Shangla	Bar Showar	September 2019	Debris	Cattle, goats	2 N, 1 F	2 N
	Jaba	September 2019	Crack	Sheep	2 N, 1 F	1 N
	Faiza	September 2019	Crevice	Equids	2 N, 1 F	2 N
	Alagram	October 2019	Crevice	Buffaloes	2 N, 1 F	2 N
	Towa	October 2019	Crack	Sheep, goats	2 N, 1 F	1 N
	Makra	September 2019	Burrow	Cattle	2 N, 1 F	–
	Kas	September 2019	Debris	Goats	2 N, 1 F	–
Bajaur	Nawagai	June 2019	Burrow	Equids, cattle	2 N, 1 F	–
	Khar	June 2019	Crevice	Goats	2 N, 1 F	–
	Wara Mamund	August 2019	Burrow	Buffaloes	2 N, 1 F	–
	Loi Mamund	August 2019	Crack	Goats	2 N, 1 F	–
Lower Dir	Ben Shahi	June 2019	Burrow	Sheep, cattle	2 N, 1 F	–
	Samar Bagh	July 2019	Crevice	Equids	2 N, 1 F	1 N
	Balambat	July 2019	Crack	Cattle	2 N, 1 F	2 N
Upper Dir	Dir	September 2019	Debris	Buffaloes	2 N, 1 F	–
	Barawal	August 2019	Crevice	Cattle, goats	2 N, 1 F	–
	Sheringal	September 2019	Burrow	Equids	2 N, 1 F	–
Orakzai	Orakzai	June 2019	Debris	Cattle, buffaloes	2 N, 1 F	–
Total					36 N, 18 F	11 N

*N* Nymph,* F* female

Abundance and percentage of the *Ornithodoros* sp. collected from various locations in the examined districts are summarized in Table 2.

**Table 2:** Abundances of different life stages of the *Ornithodoros* sp. collected in this study

Districts	Collection sites	Larvae (%)	Nymphs (%)	Males (%)	Females (%)	Total (%)
Shangla	Bar Showar	3 (0.13)	230 (9.87)	145 (6.22)	89 (3.81)	467 (20.04)
	Jaba	8 (0.35)	33 (1.41)	46 (1.97)	28 (1.20)	115 (4.93)
	Faiza	12 (0.51)	90 (3.86)	88 (3.77)	55 (2.36)	245 (10.51)
	Alagram	8 (0.34)	27 (1.15)	39 (1.67)	21 (0.90)	95 (4.07)
	Towa	14 (0.60)	161 (6.90)	151 (6.48)	198 (8.49)	524 (22.48)
	Makra	9 (0.38)	142 (6.09)	116 (4.97)	66 (2.83)	333 (14.29)
	Kas	6 (0.25)	22 (0.94)	33 (1.41)	15 (0.64)	76 (3.26)
	Total	60 (2.57)	705 (30.25)	618 (26.52)	472 (20.25)	1855 (79.61)
Bajaur	Nawagai	10 (0.42)	12 (0.51)	5 (0.21)	9 (0.38)	36 (1.54)
	Khar	18 (0.80)	14 (0.60)	10 (0.42)	12 (0.51)	54 (2.31)
	Wara Mamund	9 (0.38)	9 (0.38)	4 (0.17)	3 (0.12)	25 (1.07)
	Loi Mamund	8 (0.34)	20 (0.85)	14 (0.60)	17 (0.72)	59 (2.53)
	Total	45 (1.93)	55 (2.36)	33 (1.41)	41 (1.75)	174 (7.46)
Lower Dir	Ben Shahi	11 (0.47)	6 (0.25)	4 (0.17)	7 (0.30)	28 (1.20)
	Samar Bagh	15 (0.64)	13 (0.55)	10 (0.42)	10 (0.42)	48 (2.06)
	Balambat	2 (0.08)	12 (0.51)	8 (0.34)	12 (0.51)	34 (1.45)
	Total	28 (1.20)	31 (1.33)	22 (0.94)	29 (1.24)	110 (4.72)
Upper Dir	Dir	21 (0.91)	10 (0.42)	7 (0.30)	4 (0.17)	42 (1.80)
	Barawal	16 (0.68)	6 (0.25)	2 (0.08)	5 (0.21)	29 (1.24)
	Sheringal	6 (0.25)	22 (0.94)	11 (0.47)	16 (0.68)	55 (2.36)
	Total	43 (1.84)	38 (1.63)	20 (0.85)	25 (1.07)	126 (5.40)
Orakzai	Orakzai	3 (0.12)	21 (0.90)	18 (0.77)	23 (0.98)	65 (2.78)
	Total	3 (0.12)	21 (0.90)	18 (0.77)	23 (0.98)	65 (2.78)
Overall total		179 (07.70)	850 (36.48)	711 (30.51)	590 (25.32)	2330 (100)

### Sequences and phylogenetic analysis

**Fig. 3:**
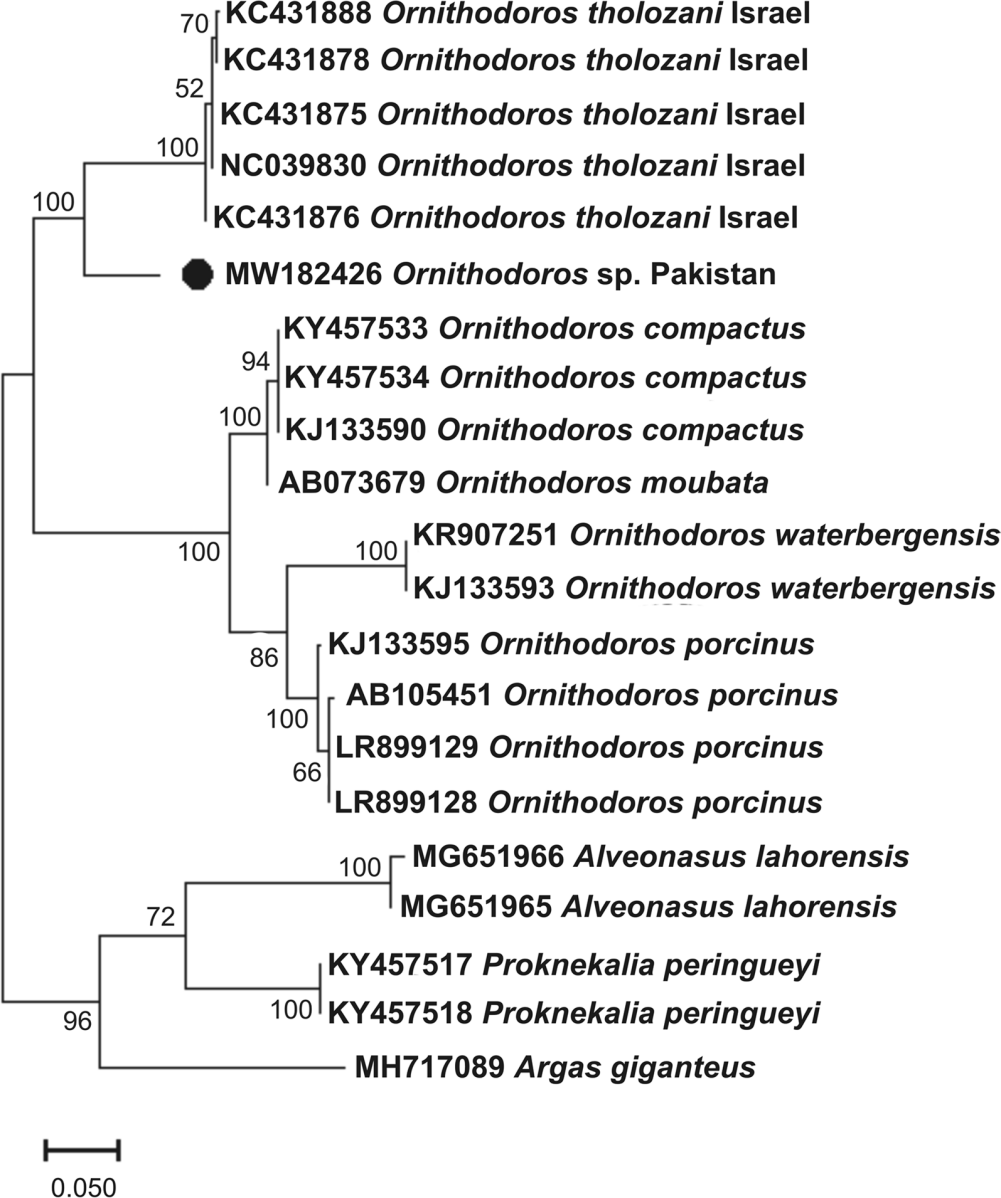
Neighbor-joining (NJ) phylogenetic tree of *Ornithodoros* spp. based on the partial mitochondrial 12S ribosomal DNA (rDNA) gene. A group formed by 12S rDNA sequences of *Argas giganteus*, *Proknekalia peringueyi* and *Alveonasus lahorensis* was employed as an outgroup. In the tree, accession numbers are followed by the species name and collection sites. The bootstrap values (1000) are shown at each node. The sequence generated in this study (MW182426) is highlighted by a black circle

**Fig. 4:**
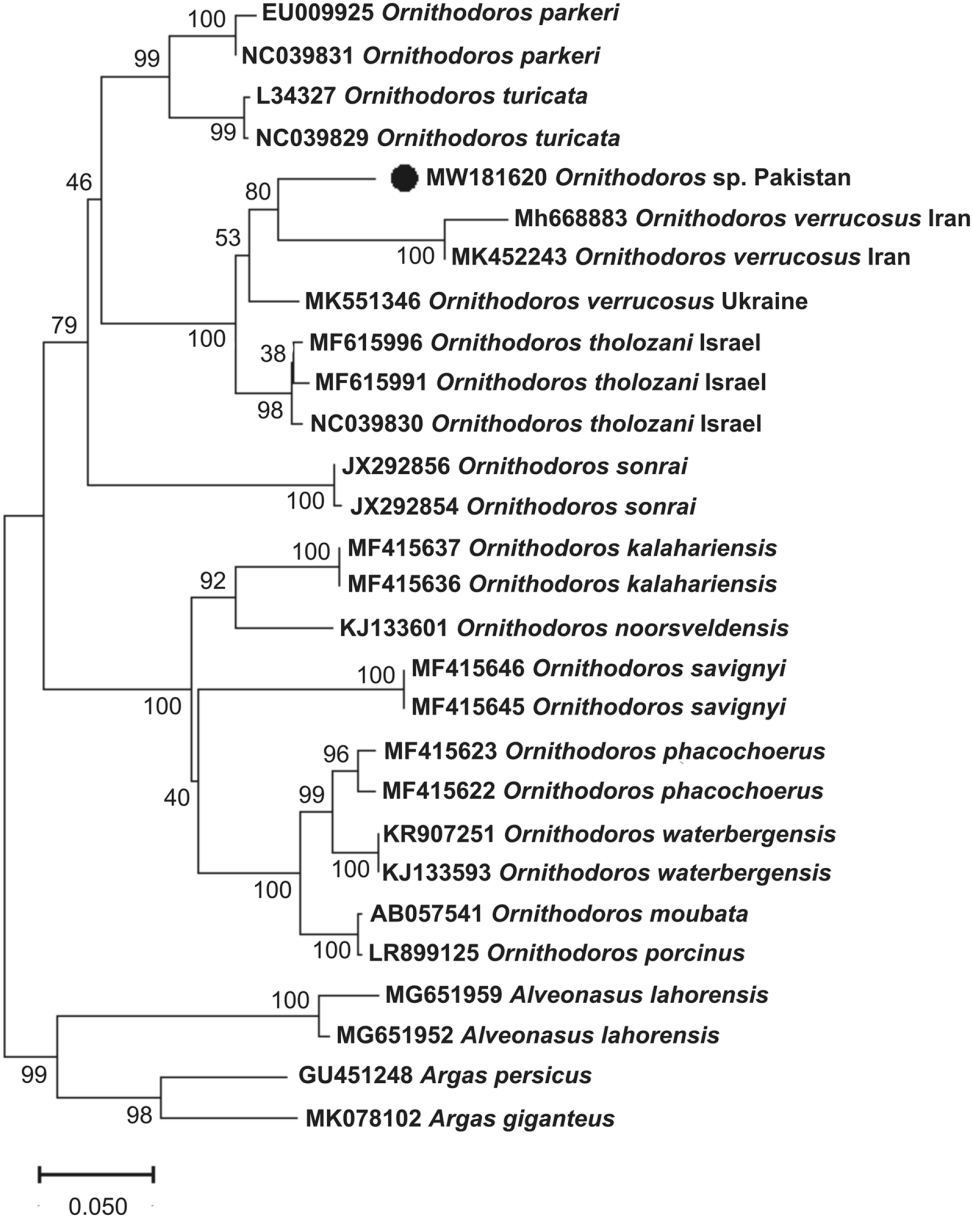
NJ phylogenetic tree inferred from the partial mitochondrial 16S rDNA gene of the *Ornithodoros* spp. A group formed by 16S rDNA sequences of *Argas giganteus*, *Argas persicus* and *Alveonasus lahorensis* was employed as an outgroup. The accession numbers are followed by the species name and collection sites. The bootstrap values (1000) are shown at each node. The sequence generated in this study (MW181620) is highlighted by a black circle. For abbreviations, see Fig. 3

**Fig. 5:**
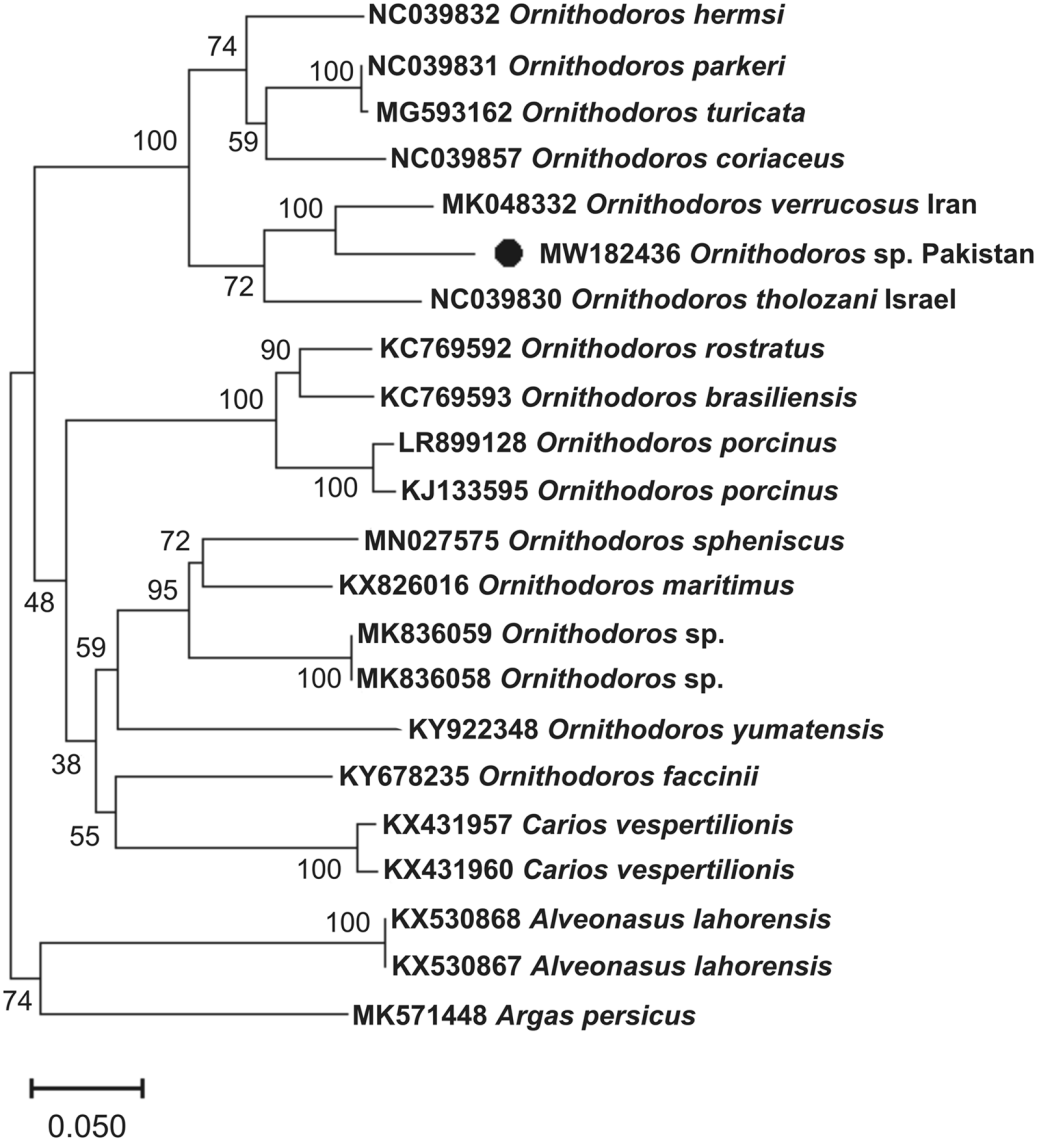
NJ phylogenetic tree of *Ornithodoros* spp. was constructed based on the partial cytochrome c oxidase subunit I (*cox1*) gene. A group formed by the *cox1* sequences of *Argas persicus* and *Alveonasus lahorensis* was employed as an outgroup. GenBank accession numbers are followed by the species name and collection point. The bootstrap values (1000) are shown at each node. The sequence generated in this study (MW182436) is highlighted by a black circle

The BLAST (NCBI) results for the 12S rDNA [336 base pairs (bp)] gene showed maximum percentage identity of 88.9% with *O. tholozani*; the 16S rDNA (383 bp) and *cox1* (639 bp) genes showed 93.5% and 90.2% identity with *Ornithodoros verrucosus* (synonym *Ornithodoros asperus*), respectively. A total of 20, 27 and 21 homologous sequences were downloaded in FASTA format for 12S rDNA, 16S rDNA and *cox1* partial genes, respectively, from NCBI. In the phylogenetic analyses (Figs. 3, 4, 5), the obtained sequences clustered with *O. tholozani* from Israel for the 12S rDNA gene; with *O. verrucosus* species reported from Iran and Ukraine for the 16S rDNA gene; and with *O. tholozani* and *O. verrucosus* reported from Israel and Iran in the case of the *cox1* gene.

**Fig. 6:**
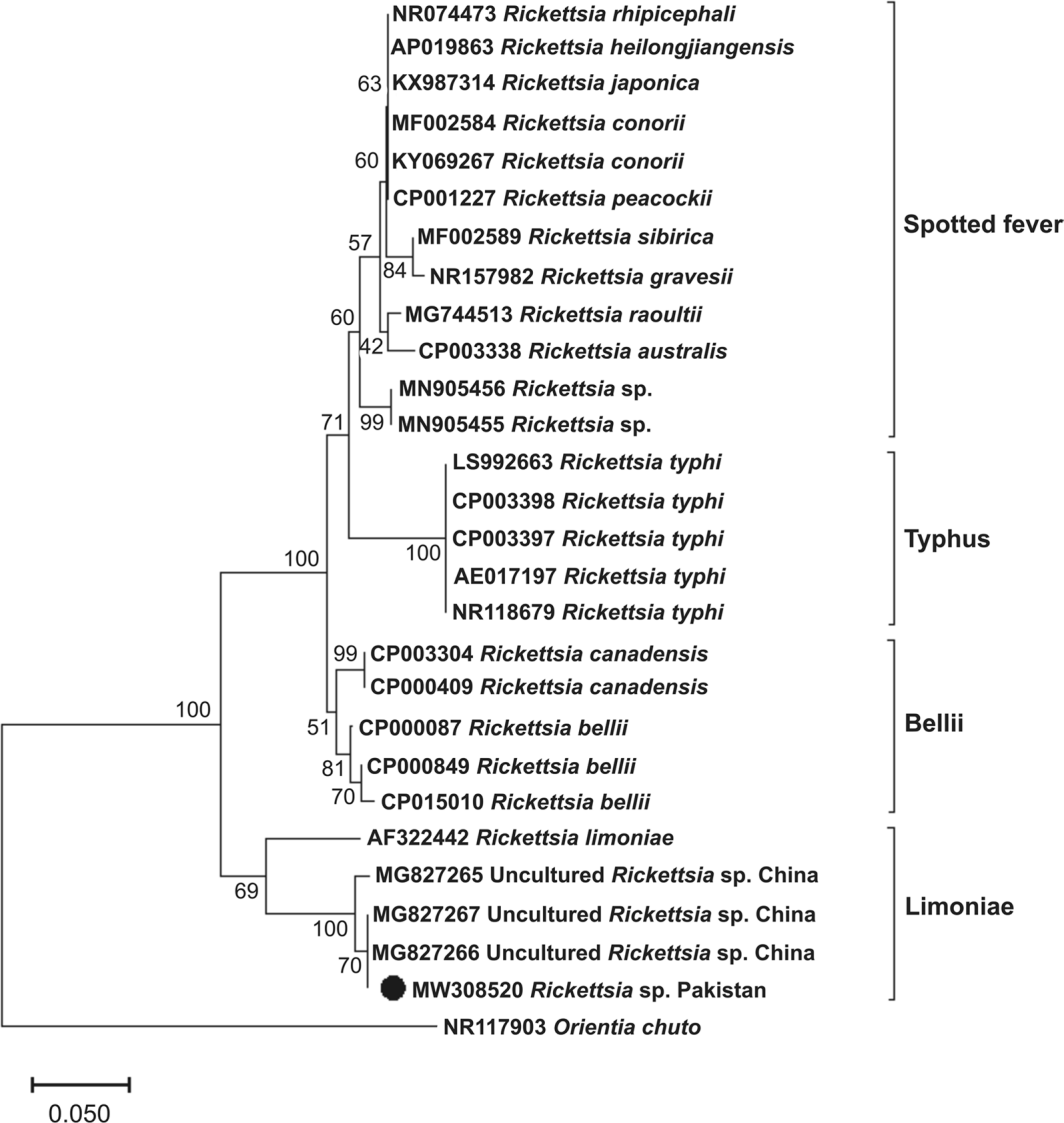
NJ phylogenetic tree of *Rickettsia* spp. based on the partial 16S rDNA gene sequence. The sequence of the 16S rDNA gene of *Orientia chuto* was used as an outgroup. The bootstrapping values (1000) are shown at each node. The position of the *Rickettsia* sp. detected in the current study (MW308520) is highlighted by a black circle. For abbreviations, see Fig. 3

Ticks collected from seven of the 18 sampling locations (five locations in Shangla and two in Lower Dir) were positive for *Rickettsia* sp., and had an overall infection rate of 20.4% (11/54) (Table 1). The BLAST results of the obtained *Rickettsia *16S rDNA (702 bp) partial gene showed a maximum identity of 100% with a *Rickettsia* sp. detected in *Argas japonicus* ticks from China. In the phylogenetic tree, the 16S rDNA gene sequence of *Rickettsia* sp. (MW308520) from the present study clustered with rickettsial sequences of the limoniae group, including those from *Rickettsia limoniae* (AF322442) and uncultured *Rickettsia* species (MG827265, MG827266 and MG827267) detected in *A. japonicus* (Fig. 6).

## Discussion

Like in many other countries, and regions of the world, the diversity of soft ticks in Pakistan has barely been explored. To the best of our knowledge, only three species of soft ticks from Pakistan have been molecularly identified, namely *Argas persicus* [33], *Argas* sp. “rousetti” and *Carios vespertilionis* [46]. In addition, five other species of soft ticks have been reported from Pakistan based on morphological identification: *Argas abdussalami* [4], *Argas reflexus* [4],* O. tholozani* [17, 18], *Argas lahorensis* [18, 19] and *O. papillipes* [19]. There is no genetic information on *Ornithodoros* spp. from Pakistan, and this lack of information needs to be addressed. Therefore, we aimed to molecularly characterize *Ornithodoros* ticks by using three genetic markers to delineate the species of this genus collected in the KP region of Pakistan. The next objective was to molecularly detect *Rickettsia* spp. in the collected ticks. The phylogenetic analysis revealed that the collected ticks are related to *O. verrucosus* and *O. tholozani* reported from Ukraine, Iran and Israel [21, 47–49]. The phylogenetic analysis of the *Rickettsia* sp. showed that it is closely related to the limoniae group, a basal group of *Rickettsia* species.

Many argasids, including *Ornithodoros* species, are nidicolous and live in sheltered habitats such as burrows, crevices, nests and old man-made shelters [6, 48, 50], and have a long lifespan of up to 20 years [51]. *Ornithodoros tholozani*, a member of the subgenus *Pavlovskyella* within the genus *Ornithodoros*, has been reported from stables, barns, stone walls, recording studios and human dwellings [4], whereas *O. verrucosus* has been reported to mainly inhabit burrows and/or crevices inhabited by reptiles and small mammals [3, 4]. These tick species are found in deserts, semi-deserts and dry areas of the Palearctic zoogeographical region, and chiefly feed on rodents of the family Cricetidae [4, 47]. In the present study, *Ornithodoros* ticks were collected from burrows, crevices, and cracks in the walls and from the ground debris of domestic animal shelters in northern and southern districts of KP. These districts generally have cold winters, mild summers and rainfall in all the seasons.

The collected ticks were morphologically comparable to *Ornithodoros* (*Pavlovskyella*), and their genetic identity confirmed this similarity since the sequences of their loci clustered with those of *Ornithodoros* (*Pavlovskyella*) specimens from Iran, Israel, and Ukraine in the phylogenetic tree (Figs. 3, 4, 5). However, it is important to note that, on the basis of the topology of the phylogenetic tree and genetic comparisons with other *Ornithodoros* ticks, the collected ticks might represent separate species to *O. tholozani* or *O. verrucosus*. *Ornithodoros tholozani* was originally described from Persia [16], an antique territory that does not include Pakistan, and the sequences presently available for this species are from Israel. *Ornithodoros verrucosus* occurs mainly in the Caucasus and eastern Europe, thus the available sequences from Ukraine are likely representative of this species [21]. In the 16S rDNA phylogenetic tree, both *O. tholozani* from Israel and *O. verrucosus* from Ukraine grouped into separate clades (Fig. 4). However, it is noteworthy that the sequences of the ticks identified as *O. verrucosus* from Iran did not cluster with those of *O. verrucosus* from Ukraine, and thus are thought to correspond to another *Pavlovskyella* species. These findings highlight the need for the accurate morphological identification of *Ornithodoros* (*Pavlovskyella*) spp. with a Palearctic distribution. Remarkably, there are only a limited number of sequences available in NCBI for *O. tholozani* and *O. verrucosus*, and our obtained 12S rDNA, 16S rDNA and *cox1* sequences showed maximum percentage identity with *O. tholozani* (88.9%), *O. verrucosus* (93.5%) and *O. verrucosus* (90.2%) reported from Israel, Ukraine and Iran, respectively. As the study of larvae has proved useful for the differentiation of *Ornithodoros* spp. [4], a taxonomic approach that considers this life stage should be used to elucidate the status of *O. verrucosus* from Iran, and also that of the *Ornithodoros* sp. collected in this study.

The nidicolous lifestyle of *Ornithodoros* species makes it challenging to develop potential mathematical distribution models for these soft ticks using correlative niche modeling [52]. It is also difficult to judge the actual relationships between their life history traits and external environment [3]. However, it has been observed that the geographical distribution of species of the subgenus *Pavlovskyella* within the genus *Ornithodoros*, which include *O. tholozani* and *O. verrucosus*, is influenced by humidity and precipitation [3, 53]. These ticks have been mostly reported from areas with microclimatic conditions marked by temperature and relative humidity ranges of 17–20 °C and 70–80%, respectively [48, 54]. Similarly, in the present study, the *Ornithodoros* specimens were collected from regions with high relative humidity, high precipitation, and high temperatures. The monthly mean temperature, relative humidity and annual precipitation in the studied districts in the summer were as follows: Bajaur—25 °C, 68%, and 700 mm; Lower and Upper Dir—22 °C, 64% and 750 mm; Shangla—20 °C, 72% and 850 mm; and Orakzai—25 °C, 61%, and 600 mm (climate-data.org). Interestingly, favorable conditions for the survival and growth of most argasid ticks, including *Ornithodoros* species, were reported to be provided by the heat and respiration generated by their host animals inside the caves in which they were found [3, 48]. Additionally, temperature has a profound effect on the activity of argasids [3], including *Ornithodoros* species [55]. We collected *Ornithodoros* ticks in the summer, from June to September, and even in this season, the specimens were never found out of their microhabitats during the daytime, which agrees with previous findings that these soft ticks are mainly nocturnal [50]. According to Hopla et al. [56], few *Ornithodoros* species that feed on livestock and poultry also feed on wild animals. Accordingly, the inspected districts in the present study were mostly rural, with an abundance of livestock (buffaloes, other cattle, equids and small ruminants).

Being an agricultural country, inhabitants of several regions of Pakistan rear livestock and poultry in animal shelters, and within or outside their houses. Soft ticks have been documented as vectors and reservoirs for several disease-causing agents throughout the world [57]. Thus, *Ornithodoros* species might pose a threat to livestock, poultry, and to people who rear livestock, in Pakistan. To the best of our knowledge, to date, there is no published report on the molecular detection of *Rickettsia* spp. associated with *Ornithodoros* species in Pakistan. Despite the fact that soft ticks are not considered natural vectors of *Rickettsia* spp., several rickettsiae have been detected in them [10, 51, 58–60]. In several regions of the world, *Ornithodoros* species are vectors and reservoirs of causal agents of tick-borne relapsing fever (TBRF) [54, 57]. In the present study, a *Rickettsia* sp. was recorded for the first time in *Ornithodoros* specimens from Pakistan. The pathogenicity of the *Rickettsia* sp. detected in the present study needs to be investigated, given the relevance of the rickettsiae bacterial group as causal agents of emerging infectious tick-borne diseases [24].

## Conclusions

The present study reports on an *Ornithodoros* (*Pavlovskyella*) sp. tick and an associated *Rickettsia* sp. of the limoniae group for the first time from Pakistan. The morphological and phylogenetic analyses revealed that the *Ornithodoros* sp. is an undetermined sister species of *O. verrucosus* and *O. tholozani*; formal identification of this *Ornithodoros* sp. is pending. The *Rickettsia* sp*.* detected in the *Ornithodoros* specimens clustered within the limoniae group of *Rickettsia* species reported from China. Further studies on soft ticks, especially *Ornithodoros* species, are essential to explore their diversity, associated pathogens, and any economic losses that they may cause.

## Data Availability

The datasets that support the conclusions are given in the article.

## Abbreviations

BLAST: Basic local alignment search tool
*cox1*: Cytochrome c oxidase subunit I
dNTP: Deoxyribose nucleoside triphosphate
KP: Khyber Pakhtunkhwa
NCBI: National Center for Biotechnology Information
NJ: Neighbor-joining
PCR: Polymerase chain reaction
rDNA: Ribosomal DNA
